# Caring for older patients with reduced decision-making capacity: a deductive exploratory study of ambulance clinicians’ ethical competence

**DOI:** 10.1186/s12910-023-00941-w

**Published:** 2023-08-09

**Authors:** Bodil Holmberg, Anna Bennesved, Anders Bremer

**Affiliations:** Department of Health and Caring Sciences, Faculty of Health and Life Sciences, Linnaeus University, Växjö, Sweden

**Keywords:** Ambulance clinicians, Ambulance service, Content analysis, Decision-making, Ethical competence, Older patients

## Abstract

**Background:**

As more people are living longer, they become frail and are affected by multi-morbidity, resulting in increased demands from the ambulance service. Being vulnerable, older patients may have reduced decision-making capacity, despite still wanting to be involved in decision-making about their care. Their needs may be complex and difficult to assess, and do not always correspond with ambulance assessment protocols. When needing an ambulance, older patients encounter ambulance clinicians who are under high workloads and primarily consider themselves as emergency medical care providers. This situates them in the struggle between differing expectations, and ethical conflicts may arise. To resolve these, providing ethical care, focussing on interpersonal relationships and using ethical competence is needed. However, it is not known whether ambulance clinicians possess the ethical competence required to provide ethical care. Thus, the aim of this study was to deductively explore their ethical competence when caring for older patients with reduced decision-making ability.

**Methods:**

A qualitative deductive and exploratory design was used to analyse dyadic interviews with ambulance clinicians. A literature review, defining ethical competence as comprising *ethical sensitivity, ethical knowledge, ethical reflection, ethical decision-making, ethical action* and *ethical behaviour*, was used as a structured categorization matrix for the analysis.

**Results:**

Ambulance clinicians possess ethical competence in terms of their *ethical knowledge*, highlighting the need for establishing an interpersonal relationship with the older patients. To establish this, they use *ethical sensitivity* to interpret the patients’ needs. Doing this, they are aware of their *ethical behaviour*, signifying how they must act respectfully and provide the necessary time for listening and interacting.

**Conclusions:**

Ambulance clinicians fail to see their gut feeling as a professional ethical competence, which might hinder them from reacting to unethical ways of working. Further, they lack *ethical reflection* regarding the benefits and disadvantages of paternalism, which reduces their ability to perform *ethical decision-making*. Moreover, their *ethical knowledge* is hampered by an ageist approach to older patients, which also has consequences for their *ethical action*. Finally, ambulance clinicians show deficiencies regarding their *ethical reflections*, as they reflect merely on their own actions, rather than on their values.

**Supplementary Information:**

The online version contains supplementary material available at 10.1186/s12910-023-00941-w.

## Background

Populations are ageing worldwide, and the number of people aged 80 or above is expected to triple between 2020 and 2050 [1]. As people age, their bodies and minds become worn, thus weakening in a natural way to become frail [2]. This results in a lowered bodily, mental, and social resistance to deal with strain and stress. Adding to this, many are affected by multi-morbidity and a high symptom burden that further limits their executive capacity and makes them vulnerable [3]. At high age, heart failure, cancer and major neurological disorders are common [4], and these are often combined with varying degrees of dementia, a disease that increases in parallel with longevity and causes loss of cognitive abilities, dependency and impaired decision-making capacity [5]. Despite this, older people usually value being involved in decision-making about their health care [6], and the sense of having control over their own life contributes to dignity in the midst of vulnerability [7]. This includes those older people who independently choose not to exercise control and transfer their decision-making to trusted others [8].

The rising prevalence of older people affected by multi-morbidity has been ascribed to increasing demands on ambulance services [9]. Older patients’ vulnerability makes them potentially difficult to assess in acute care, as their needs are complex, thus not only medical, but also psychological, social and existential, in combination with a lowered personal capacity to act appropriately on their own behalf [10]. This group of older patients constitutes a significant proportion of non-convey patients, as they are often classified as having non-specific complaints that do not fit with the ambulance clinicians’ (ACs) assessment protocols [11]. ACs, who consider themselves as providers of emergency medical care working under high workloads of dispatch calls, have described themselves as being in a struggle between differing expectations when they disagree with the older patients’ request for emergency care, while also enjoying taking an interest in their problems and in providing them comfort [12]. The ACs’ inner struggle conveys a risk of lacking empathy and seeming rude to the patients. Thus, ethical conflicts may arise when there are different care options and the older patients’ best interest is difficult to discern, or when ACs, older patients and bystanders disagree on the emergency care needs [13].

As older vulnerable patients have a lowered capacity to defend and protect their rights, they are at risk of abuse and neglect by the healthcare professionals, who hold the power in an asymmetrical patient relationship [14]. Thus, in nursing practice, such as in ambulance care, ACs must adhere to the four cornerstones of biomedical ethics by respecting patients’ autonomy, promoting beneficence and nonmaleficence, and striving for justice [15]. In practice, ethical care is performed within an interpersonal patient relationship that builds upon respect and benefits the well-being of both ACs and patients [16]. This promotes caring relationships that constitute the foundation of ethics. To achieve this, ACs need to possess ethical competence, broadly defined as consisting of ethical sensitivity, ethical knowledge, ethical reflection, ethical decision-making, ethical action and ethical behaviour [17]. These concepts are distinct, though closely related to each other. For instance, ethical action depends on how the carer relates to the other dimensions of ethical competence.

In summary, as more people become old and frail and are affected by multi-morbidity, this results in increased demands for the care provided by the ambulance service. Due to their vulnerability, older patients may have reduced decision-making capacity, despite still wanting to be involved in decision-making about their health care. Their needs are complex and difficult to assess, however, they do not always align with ACs’ assessment protocols. When in need of an ambulance, older patients meet ACs who are under high workloads, and who primarily consider themselves as providers of emergency medical care. This places ACs in a struggle between differing expectations, and ethical conflicts may arise. To resolve these, ACs need to provide ethical care that focusses on interpersonal relationships and by using their ethical competence. To our knowledge, it is not known whether ACs possess the ethical competence required to provide ethical care. Thus, the aim of this study was to deductively explore ACs’ ethical competence when caring for older patients with reduced decision-making ability.

## Methods

### Design

In compliance with the aim, a qualitative deductive and exploratory design was used to analyse dyadic interviews with ACs.

### Setting

The publicly funded healthcare system in Sweden is divided into 21 healthcare regions, which organize the ambulance service based on conditions and needs in each region. The number of ambulance stations and their location differ between the regions, based on variations in population density and geographical conditions. The study was conducted in a southeast Swedish region where the ambulance service provides care for approximately 203 000 inhabitants in rural and urban areas on a total of 8 458 km^2^. The overall population density was 24/km^2^. The region had eight ambulance stations with a total of 17 ambulances providing Advanced Life Support (ALS). The ambulances were staffed with two ACs, at least one of whom was a specialist ambulance nurse (SAN).

The education and training to become a SAN comprises a 1-year master’s degree and a postgraduate diploma in specialist nursing for registered nurses (RNs). In order to qualify for the programme, students must be registered as an RN with a Bachelor of Science degree, including specialisation in caring or nursing science. The staff within Swedish ambulance services consists of specialist trained registered nurses, registered nurses (RN) and emergency medical technicians (EMT). Each ambulance must be staffed by at least one licensed AC. In 2021 the Swedish ambulance service consisted of 53% specialist trained RNs, 28% RNs, and 19% EMTs. SANs and other specialist trained nurses have undergone a 4-year university education, i.e., three years to become RN and one year for specialist education. EMTs typically have 2 years of high school nursing education, which is supplemented by a 6 month to 1 year ambulance care specialization. In the present study, the distribution in the participating region was 78.5% specialist trained nurses (dominated by SANs), 15.5% RNs, and 6% EMTs. The total number of employed ACs was 148 [18].

Patient care in the Swedish ambulance services generally focuses on the patients’ physical and biomedical status by using the A-E principle (airway, breathing, circulation, disability, and exposure), observing vital signs, and listening to the patient’s perceived symptoms of illness. Most ACs use the Rapid Emergency Triage and Treatment System (RETTS) to assess the patient’s medical care needs. In addition to these general guidelines, there are a number of clinical practice guidelines and concepts for assessment, prioritization and treatment of specific situations and conditions. Among these can be mentioned guidelines for home-based self-care, non-conveyance of the patient, prehospital trauma life support, advanced medical life support, major incident medical management and support, prehospital medical management, and ongoing deadly violence [19].

### Participants

The participants were recruited based on a convenience principle. Employed ACs (*n* = 35) were informed about the study by the second author in a staff meeting or by their head in regular staff meetings. Those who showed interest but did not participate in the staff meetings were contacted by the second author. The inclusion criteria were clinically active ACs with professional affiliation as RNs with or without specialist education in ambulance care, anaesthesia care, or intensive care, or as EMTs.

In total, the 30 ACs who agreed to participate were assigned to constellations of 15 interviews. Participating EMTs (n = 4) had a mean age of 57 years (range 47–65) while the RNs’ (n = 26) mean age was 41 years (range 30–56). The work experience of the EMTs was 31 years on average (range 16–40), while the RNs had an average of 11 years of work experience (range 1–28).

### Data collection

Dyadic interviews were chosen to give participants the opportunity to share their thoughts and feelings with each other during the joint interview. Dyadic interviews aim to examine the participants’ experiences when brought into dialogue, and contribute their perspective to the whole. This enables researchers to capture nuances and characteristics in a way that is difficult in individual interviews [20]. The idea of dyadic interviews in the present study was also to mimic the ambulance team and reach similar discussions in the dyad. Some dyads were made up of the ambulance team that normally worked together, while other dyads were put together only for the interview occasion. Interview data were collected between December 2019 and February 2020, by use of a case vignette technique, that is, providing short descriptions of situations with specific circumstances for participants to reflect upon [21]. This method is relevant when studying professionals’ actions, as it generates knowledge of their ideas, explanations, values, norms and ethical positions [22]. A three-step case vignette, based on an emergency prehospital situation mirroring an ethical dilemma, was used (see supplementary file, Table S1). The vignette was constructed specifically for the present study and was based on literature reviews, methodological literature, and a critical review of the authors’ experiential knowledge.

The vignette was presented following a joint structure in all interviews, where steps two and three of the vignette were presented to the participants when their narratives subsided. Open-ended follow-up questions were posed, such as: “How do you assess the patient’s decision-making ability”? Follow up questions were asked to elaborate further on the ACs’ understanding of older patients’ self-determination when caring for those with reduced decision-making ability. The interviews (*n* = 15) were recorded and lasted 35–77 min (mean = 61 min). They were performed by the second author and transcribed verbatim by a professional transcriber.

### Data analysis

This study is a secondary analysis of a rich dataset. A primary analysis with an inductive and meaning-seeking thematic approach will be published elsewhere. A deductive content analysis [23] was performed, starting with the first author listening to the interview recordings and reading transcripts to obtain a sense of the whole and become familiar with the data. A literature review by Lechasseur et al., including 89 articles defining ethical competence in the context of nursing practice [17], was used to construct a structured categorization matrix. The concepts of ethics in nursing defined in the review then formed the main categories, while explanatory concept-related text drawn from the review was used to generate headings for the sub-categories (Table 1).

**Table 1:** Matrix of analysis

Ethical competence				
**1. Ethical sensitivity**	a) Evaluates and interprets reactions and feelings	b) Interprets needs based on what is said and behaviours	c) Uses compassion	d) Identifies ethical problems
**2. Ethical knowledge**	a) Has relational knowledge – emphasizes the importance of mutuality, relationship and curiosity	b) Has knowledge of embodiment – emphasizes the importance of dealing with bodies as lived subjects	c) Has philosophical, theoretical, and practical knowledge	d) Has contextual awareness and lay knowledge
**3. Ethical reflection**	a) Reflects on ethics in consideration between courses of action	b) Reflects on the own role and task as ambulance carer	c) Has an internal reasoning that clarifies own values	d) Balances risk–benefit based on prioritization, equality and morality
**4. Ethical decision-making**	Undergoes a process that leads to a reasonable and responsible choice between several options			
**5. Ethical action**	Acts on the basis of knowledge, reflection, analysis and decision-making			
**6. Ethical behaviour**	a) Shows respect for others	b) Behaves masterfully and moderately	c) Is responsive	d) Confirms the other’s concerns

A search for content that corresponded with the matrix’s main categories and sub-categories was performed, and the extracted data were placed in datasheets. The categorization of the data followed the structure of the matrix, resulting in the development of the six main categories and sixteen sub-categories presented below. The first author performed the analysis.

## Results

### Ethical sensitivity

*ACs identify the limited time spent with older patients as an ethical problem that may result in actions that overrule the patients’ explicit desires and caring needs. To counteract this, they interpret the patients’ needs by compassionately observing and listening.*

a. ACs evaluate older patients’ glances and gestures to note whether there is an immediate and positive connection or whether the patients reacts by withdrawing. If the patients withdraw when touched, it is tentatively interpreted as unwillingness to accompany the ambulance and the declining of care. Also, the reactions of significant others and care staff are observed to assess whether ambulance transport was ordered in consultation or was a unilateral decision. If the patients remain passive, this may be interpreted as having given in to the pressure of others.

b. Older patients’ needs are primarily interpreted from what they tell ACs. Thus, the AC’s initial questions are open-ended and aimed at obtaining the patients’ own description of their condition, but also to assess their cognitive status. If impaired cognition is obvious, ACs turn to significant others or care staff to interpret the patients’ needs. The patients’ behaviour is noted and serves as a guide in the assessment but needs to be verbally confirmed to be considered reliable. The behaviour of bystanders is also interpreted and contributes to the assessment, for example if there is great urgency in packing personal belongings to send with the patients.

c. ACs use their compassion to assess the situation. They empathize with the older patients and try to imagine their situation of weakness and vulnerability, understanding that their ability to make their will heard may be limited. They therefore side with the patients and their right to make independent decisions, even if it may mean that they refuse care.

*I think you have to respect her will, if it’s the care she doesn’t want. That she wants to finish. Maybe she’s tired of her suffering ... she’s certainly been in hospital a lot, in and out … (Interview 4)*

ACs feel sorry for older patients who are transferred to new environments at inconvenient times while suffering from symptoms that may be exacerbated by the transition. Likewise, they experience uneasiness when carrying out treatments that may harm the patients’ bodies. Similarly, ACs describe having a guilty conscience when they provide CPR to patients whom they deem to have little chance of survival.

ACs understand that significant others can react negatively based on a lack of healthcare experiences, internal conflicts when receiving less information than others, or lack of insight due to making few visits to the patient. Significant others’ desire to keep their loved one alive can explain their inability to accept the patient’s deterioration, thus urging the ACs to do everything in their power to save these patients’ lives.

d. ACs identify the short time they spend with the older patients as an ethical problem that hampers their ability to determine the patients’ cognition and decision-making ability. This becomes more difficult when the patients are so lacking in consciousness that they cannot express their will. ACs turn to significant others or care staff, which can then lead them to transporting the patients to hospital against their will. This problem can be accentuated when patients are dying and communication about former care decisions is lacking:

*I had no idea there was a decision on palliative care and so we just went in and demolished what patient and doctor planned about fourteen days ago. I think that’s hard. (Interview 6)*

This can mean that dying patients are subjected to CPR and the stress of being transported, despite having a Do Not Attempt Resuscitation (DNAR) order.

### Ethical knowledge

*ACs mention ethical concepts, but do not elaborate on their meaning. Rather, they rely on their own personal experiences of how older people function. Conversely, they also possess knowledge about contextual possibilities for care, and the value of establishing a mutual relationship that clarifies the patients’ perspective.*

a. ACs describe how it is important to create a mutual and intimate relationship with older patients, partly to ascertain their cognitive status, but above all to gain knowledge about their personal wishes. Thus, ACs initiate a dialogue to lower the influence that their own interpretations may have on the situation. In conversations, it is important to listen and be sensitive to the patients’ thoughts and experiences. To achieve this, ACs ask investigative questions to encourage storytelling and patient participation. In such conversations, the patients’ trust is expected to grow. Meanwhile, privacy is assured by the colleague who asks others to leave the room. ACs also pose general questions about other things, as this is believed to make the patients relax. At times, making special arrangements are beneficial:

*I proposed we should have a cup of coffee and sit down and reason a little bit about this. And we did. And all of a sudden when you sit with a cup of coffee, everything becomes much easier to solve. Then you break down these roles, the ambulance roles, the family roles and the role of the patient, you become involved on a completely different level. (Interview 7)*

If the patients have difficulties with speaking, ACs talk to significant others or staff who may know the patients well. In all conversations, it is important to be clear and explain until everyone has understood what options exist to resolve the situation.

b. ACs imagine the outside world from the perspective of the patients, which makes them understand that older, ill patients do not always have the strength to make their will heard in competition with the voices of the strong and healthy. ACs describe that there is a risk of making the patients feel anxious when they are being transferred to an ambulance, then further transported to a hospital, where they are usually unfamiliar with both the context and the people surrounding them. Patients who are cared for at home are believed to find it easier to be self-determining, as home is most often perceived as a safe place.

c. Taking life-saving measures with multi-diseased and dying patients, followed by strenuous ambulance transport to hospital, was described as being meaningless, only serving to increase the patients’ suffering. Treatment can alleviate suffering, but so can limiting the amount of care provided. Transporting patients to hospital against their will was described as an assault, as the patients’ statutory right to exercise self-determination over their own body and life then is ignored. Additionally, ACs risk violating the patients’ dignity when exposing their bodies to heavy-handed treatment, i.e., CPR.

d. ACs take the environment on site into account when making conclusions, i.e., when observing an older patient who receives help with oral care and concluding that this person is at the end of life. Likewise, they have a contextual awareness of other professionals’ competence. Once a palliative care team is involved, ACs assume that the patients have planned to die at home, surrounded by a multi-competent team of registered nurses, doctors, and care staff, who possess the same medical resources found in hospitals, and are able to explain end-of-life issues to significant others. In home care, there is a lack of time for providing such attentive care, as municipal registered nurses are responsible for a large number of patients. However, the municipal home care staff is expected to have good knowledge of how to provide good basic care, and thus easily be able to continue care when ACs have relieved any acute problems. When acute illness occurs in nursing homes, care staff are often perceived as insecure and uninformed, which explains why they call for an ambulance. However, ACs think that it may be better for patients to remain at the nursing home, as there is access to drugs and round-the-clock monitoring. Moreover, ACs have lay knowledge based on their own personal experiences, knowing that older patients often become confused and distressed when hospitalized. Further, older people are assumed to come to a point when they peacefully accept that life is over and prefer to die calmly at home. ACs experienced that measures with good intentions taken against the patients’ will can have negative consequences:

*When mom had cancer, they wanted to take a biopsy on the tumors ... She let them do it, but didn’t want them to take anything away, because she’d rather die earlier and keep her quality of life. There they overruled her, because they picked off tumors anyway. Then she wasn’t clear in her head anymore and that feels bitter now. After all, they did their best, thinking that there was nothing to lose. But there was ... (Interview 3)*

Therefore, ACs advocate that one should always comply with the wishes of the older patients.

### Ethical reflection

*ACs’ internal reflections primarily concern their own actions, not their values. They constantly consider risks and benefits to older patients, but also regarding themselves. In their reflections, their organization is medical-oriented, but with time and experience they can earn the courage to make more holistic nursing assessments.*

a. In situations where older patients are acutely ill or have suffered cardiac arrest, ACs make ethical considerations regarding the need for CPR and hospital care. Some ACs say they lack choice, as their guidelines are strictly medical. Therefore, they treat and transport older severely ill or dying patients and provide CPR even if it feels wrong. Other ACs believe that older patients’ self-determination is superior to the guidelines and refrain from providing, or discontinue, treatment when necessary to protect the patients’ dignity:

*I was given a regular transport, a patient with breathing problems, and found an older man with Cheyne-stokes breathing. I said: ‘We can’t drive him in this condition, he can die on the road!’ We stopped and held his hand while he died peacefully. I have no problem making such decisions, because I look at the ethical. We do not have eternal life, that’s important to remember. Not fight to keep them alive but let them finish in a good way. (Interview 7)*

b. When reflecting on their role, ACs describe themselves as older patients’ advocate. They have been called to the site for their sake and are therefore prepared to fight to defend their will. At the same time, their mission is to save lives, work quickly, and transfer patients who sometimes have long transport times to hospitals. They are trained to solve problems, to start from metrics and to follow guidelines instead of valuing the patients’ quality of life. In the ACs’ reflections, the ambulance culture values medical knowledge more highly than nursing knowledge. Therefore, it feels wrong not to transport the patients to the hospital when someone has raised the alarm.

ACs describe themselves as having authority, based on their competence and education. This means that their medical assessments are more knowledgeable than those made by older patients, significant others and other staff. In their reflections, this is why they are not always responsive to patients’ wishes, but instead persuade them into complying with their own suggestions. Sometimes, ACs stand between the will of the patients and their significant others, which makes decision-making difficult. However, deciding whether the patients are going to hospital or not, is occasionally considered the work of others:

*This lady has a nurse from the palliative team who looks after her. And I think, they have to make that decision! We wouldn’t even have to interfere in it, but be able to say: ‘We’ll wait outside, and you’ll tell us when you’ve decided whether or not she’s going to join’. (Interview 10)*

At the same time, this is described as being a difficult situation, where ACs are afraid to make mistakes, and risk being reported and lose their right to practise their profession. They consider it their duty to inform about alternative solutions to facilitate informed decisions, but, in uncertain situations, they prefer to take the patients to hospital to protect themselves from disciplinary actions.

In the ACs’ reflections, despite prioritizing medical assessments, the ability to make holistic nursing assessments can grow over time and make an AC confident enough to refrain from CPR, to question doctors, and take sides with older patients who want to stay at home. Experienced ACs possess both medical and nursing experience that recent graduates often lack, therefore, those with less experience must rely entirely on clinical metrics. Thus, there is a desire for ACs to be given a greater scope for making nursing assessments in practice, and they also describe that having more knowledge about multi-diseased older patients and ethics is necessary.

c. ACs describe feeling ambivalent about decisions they have made when choosing to follow older patients’ will, contrary to their guidelines. In their internal reasoning, they ask themselves what benefits alternative measures could have had. In some cases, they defend paternalism because the patients did not understand their own best interests, and in other cases, they regret their actions:

*Who am I to decide when people should receive care or not, if they are not at the full use of their minds? I can’t decide who will live or die. (Interview 6)*

In order to prevent negative consequences, ACs carefully document their own actions and the patient’s wishes in the medical record when their actions deviate from medical guidelines stating that the patient’s condition should be assessed and treated in hospital. In cases when they are convinced that the decision is in the patient’s best interest, they may even adapt the documentation to protect themselves against disciplinary actions.

Having conversations with other colleagues facilitates important reasoning that helps ACs to be satisfied with their actions. Having a discussion and mutual planning care is initiated as soon as ACs receive the initial information about the assignment, which creates a common approach. When meeting older patients, there is a continued discussion about what measures are judged to be best. Establishing unity between colleagues provides important emotional support, where a glance or a nod may be enough. After completing the assignment, it is valuable to talk it through together to confirm that decisions were appropriate. Sometimes colleagues cry together. Therefore, openness, lack of prestige and honesty are described as being important prerequisites for being able to foster professional development in a context where you are thrown between extremes on a daily basis.

d. ACs constantly consider the risks and benefits of their practices to older patients and balance these against the needs of society, especially a parallel or possible need in patients with more serious conditions than those presented by the older patient to whom they have come. To older patients, it is not considered useful to be transferred to hospital at the end of life, as they often have long waiting times, just to be sent back without any care measures being taken. According to ACs, hospital transports for older patients causes nothing but strain:

*I probably put more effort into persuading a 70-year-old than a 90-year-old to come along. A 90-year-old does not survive abdominal surgery in the same way, for example. The odds aren’t quite as great. (Interview 4)*

Furthermore, ACs believe that the palliative care older patients receive in their homes is equivalent to hospital care. From a societal perspective, ACs carefully consider older patients’ need of hospital care, as their stay burdens the economy and adds to the staff’s workload. Additionally, when older patients are admitted to hospital, they occupy hospital beds that could have been used for other patients with greater needs. Finally, transporting older patients to and from the hospital means that the ambulance is not available for emergency situations. Thus, ACs prefer to solve the situation by relieving the patients’ symptoms on site and coordinating the continued care with other actors.

### Ethical decision-making

*In the process of choosing between several options, ACs rely on their routines and professional experience. In relation to others, this may entail the provision of information, or persuasion in favour of ACs’ opinions and values.*

In situations with multiple possible courses of action, ACs undergo a process to devise a reasonable and responsible alternative. Initially, older patients are visually assessed upon the ACs’ arrival. These impressions are processed and compared to previous experiences to provide an overall picture of the situation. If an older patient has suffered from cardiac arrest, decisions are made in hasty consultations with present significant others or staff, to assess the patient’s prognosis. When the patients can communicate, questions are asked while the patients are being examined. In the event of reduced communication ability, ACs turn to significant others or care staff on site, or contact doctors to get more information about the patients’ status and anamnesis, whether previous decisions about palliative care at home have been made and if there is a DNAR order. When the patients want to stay at home while those around them want them to go to hospital, ACs can take the patients’ side:

*Patients are always subordinated. This can be used to their advantage, as we still have our experience with us. Many times relatives listen when we explain that they will do nothing about this in the hospital, it is better for her to stay at home in her bed. Call the health care centre tomorrow instead. (Interview 10)*

If patients, significant others, or care staff have opposing views, ACs try to facilitate decision-making by clearly informing them about possible consequences, with the ambition to create an in-depth understanding of the situation. When ACs leave the site without the patient, they make sure that they have provided contact information for another, more suitable, healthcare provider.

### Ethical action

*For ACs, ethical action merely means to provide needed care at site and avoid unnecessary actions. They do not talk about care in the ambulance or at the hospital in terms of ethical action.*

AC’s actions are sometimes about doing nothing at all, for example when an older, multi-diseased person has suffered cardiac arrest. This can cause ACs to make a decision above their authority, but one which is still deemed necessary, as the patient’s life is over. At other times, it is about mediating, reasoning, or making a stand with significant others or doctors who want to act against the patients’ will. When the patients’ status is difficult to assess and the available information fails to allow for this mediation, ACs take the patients to hospital for medical assessment. In situations where the patients are competent in making decisions and refuses ambulance transport, or has previously made a decision to be cared for at home, ACs strive to provide symptom relief on site:

*If there is something you can do at home, it is better to take care to them, than them to the care. (Interview 2)*

The patients are then left at home in accordance with their own wishes, which ACs describe as being an obvious measure.

### Ethical behaviour

*To behave ethically, ACs trust an inarticulate, intuitive gut feeling that helps them perceive nuances in other people’s demeanour. They show respect by use of body language, listening and allowing interactions with others to take time.*

a. Showing respect for others means respecting the will of older patients, which is largely about how ACs behave on site. Sitting down with the patients to have a conversation instead of standing up near the entrance door means showing respect. Similarly, ACs show respect by staying and conversing for a while, even with patients who decline transport to hospital. It is described as being respectful to clearly convey to the patients when ACs turn to significant others for more information, instead of doing it secretly. In addition, ACs show respect for the patients by marking verbally when significant others are assertive:

*When entering a room, the patient often lies down and the relatives are in one’s ear all the time. Sometimes you almost can’t think because they talk so much. And then I can say a little demonstratively: ‘Thank you very much, but now I want to listen to NN and hear what he says.’ You might step on someone’s toes a little bit, but the most important thing we have is to stand up for the patient. (Interview 12)*

Thereafter, ACs show respect to significant others by listening to them, too. This respectful behaviour also applies to colleagues in other settings, as ACs sometimes rely on their assessments rather than making their own.

b. Behaving in a controlled and moderate manner is about being courteous towards older patients, significant others, and care staff, i.e., when proposing a different solution than theirs. To lay the foundation for wise joint decisions, ACs convey security to those they meet and start conversations on an equal level. This often requires time, regardless of ACs’ stress, hunger or tiredness. In such conversations, ACs talk in ways that are assumed not to harm or frighten, while giving the other party time to realise the situation. It is described as being important to dare to initiate a conversation, even if the situation is unpleasant, with aggressive responses. If patients or significant others behave agitatedly, ACs avoid conflicts by taking a step back and allowing space for emotional reactions.

c. Being responsive is about perceiving the atmosphere in a room, for example, if something indicates an ongoing conflict. It is also about noticing colleagues’ reactions, as they may have perceived something that needs to be considered. Responsiveness also means to sense the older patients’ state of mind to understand whether the patients present a genuine wish or if they only want to please someone else. ACs describe having an ability to interpret the patients’ silence, as approval, resignation or despair. Sometimes they sense that the patients want to convey something unspoken. ACs find this capacity for responsiveness difficult to put into words, but describe it as an intuitive gut feeling:

*I couldn’t decide what he wanted, it was just a feeling I got. It’s not always, but in many cases you become sure then. Many times you should trust this feeling, what feels right in your heart. You shouldn’t belittle that, because that’s often what makes it good. It is not always possible to follow a rulebook. (Interview 12)*

d. ACs confirm the concerns of older patients and significant others by answering questions, thus signalling that their worry is understandable. Many become calmer as soon as ACs arrive on site, which is reinforced by an examination of the patient or an assurance that measured values are normal. The concerns of older patients may be due to memories of being treated rudely at the hospital. ACs then alleviate their concerns by promising to report this on arrival, thus preventing it from happening again. When patients are left at home, ACs sometimes offer to return for a supervisory visit, or reassure the patients that they are welcome to call again:

*Just because we’ve been there once, the door isn’t closed for good so you can’t call any more. It’s not like that. You have the opportunity to come back if it gets worse, or if something new should happen. (Interview 10)*

Alternatively, ACs help the patients to arrange an appointment at the healthcare centre to make them feel calm when they leave. To alleviate the worries of significant others, ACs carefully explain what they have done and what measures they have taken to examine the patient. In this way, significant others are actively participating in the care, which is designed to have a calming effect, especially when the patients are seriously ill.

## Discussion

The results show that ACs possess ethical competence, used when caring for older patients with reduced decision-making ability. This competence is primarily characterized by *ethical knowledge* regarding the importance of a relationship, which manifests itself in a desire to investigate what the patients want and adapt care accordingly to safeguard the patients’ self-determination. This aligns with an earlier study, showing that ACs attempt to respect older patients’ self-determination by collaborating with them [24]. Such promotion of patient participation in the planning of their own care has been described as an indicator of competent nursing [25]. To promote a trusting relationship, ACs describe the importance of listening to the patients and study their reactions to also perceive the unspoken. This creates conditions for a caring encounter, based on presence, recognition, availability and mutuality [26]. Within such a trustful relationship, a carer and a patient can perform an authentic and honest dialogue that creates a space of togetherness that leads to mutual well-being. To make sure of the older patients’ genuine will, ACs use *ethical sensitivity*, which helps them interpret the patients’ needs by listening to what they say and by observing how they and other people nearby behave. In this, ACs describe themselves as being the patients’ advocate, despite a constant time pressure, and regardless of their own stress, hunger or tiredness. The ACs’ focus on trustful relationships and overall responsibility can be described as being a holistic care that views older patients as biopsychosocial beings who need to be included in the planning and decisions about their own care [27].

In order to create security and enable an open communication, *ethical behaviour* is shown through respect, courtesy and control, even in situations when ACs are treated aggressively. According to Lechasseur et al., respectful behaviour is an important dimension in holistic care and a sign of a developed patient-carer relationship [17], which confirms the ACs’ ethical competence. As part of this *ethical behaviour*, ACs describe themselves as working from a gut feeling that helps them to navigate emotional situations. They find it difficult to put this ability into words, but it can be described as an ethical ability to intuitively be touched by the feelings of others and as being able to identify with their distress [15]. This competence is, according to the International Council of Nurses [28], expected to be held by all professional nurses, who are also expected to use this skill to contribute to ethical organizations and to question unethical ways of working. Interestingly, the ACs in this study, who *do* question their own organization’s focus on medical assessment and care measures and desire a greater scope for holistic nursing judgements and more knowledge of ethics, *do not* define their own ethical ability as being the fruit of performing professional practices. This reveals shortcomings in their holistic perspective that may hamper their ethical competence. Consequently, not acknowledging ethical competence as a form of professionalism may hinder them from reacting to unethical ways of working.

ACs tend to possess a degree of *ethical competence*, both in respecting the patient’s autonomy and making decisions in line with the patient’s best interests. In decision-making for the patient’s best interests, some describe themselves as having the authority, based on experience and knowledge, that entitles them to also persuade patients and significant others who do not want to follow their recommendations. This might be beneficial, as an AC who trusts in his/her own competence and experiences is more likely to gain the patients’ trust and may find it easier to apply a caring approach [29]. Alternatively, this attitude can be described as a risky exercise of power or paternalism, that is, to prevent the patient from having a choice on the basis that this will not be in the patient’s best interest, grounded in the assumption that the patient cannot make a well-considered decision [30]. This can be said to reveal shortcomings in some ACs’ *ethical reflection* that leads to problems with *ethical decision-making*. ACs’ paternalistic attitudes can be questioned as, according to the study results, they occasionally base their reasoning upon their own personal experiences of how older people function and think. This indicates a lack of *ethical reflection*, as ACs in this study appear to consider it always wrong to convey patients who do not want to go to hospital, thus it is wrong to persuade them. This raises the question of whether paternalism is always wrong. As shown by Nordby [31], ACs can use their knowledge and experience to look forward in time and assess possible outcomes, in ways that the patients cannot. Thus, if they foresee that compliance with an upset patient’s wishes may convey future health risks, their persuasion can in fact be understood as respecting the patient’s autonomy, particularly if the patient would otherwise agree after having regained a more sober perspective at a later and less acute stage. This way of protecting patients form the harmful consequences of following their involuntary choices can be termed as soft paternalism [30]. As actions in themselves cannot be paternalistic, one should look at the *motive* behind them. Hence, when the motive is respectful and aimed to protect patients from future harm, one may conclude that not all persuasion is paternalistic in a way that threatens the patients’ autonomy, dignity and integrity.

An aspect indicating a lack of *ethical knowledge* and *ethical reflection* concerns the views and preferences of older patients. ACs in this study seem to assume that older patients with impaired decision-making ability feel best about being cared for in their home, not in an ambulance or in a hospital. This is questionable, as other studies indicate that ambulance service assignments frequently involve older persons from the age of 65 and older [32], which means that the age group can include two generations, with widely different life experiences and wishes for their care. Further, ACs in this study prefer to provide care on site and avoid taking older patients to hospital, regarding their hospital stays as often unnecessary, and a burden to society. This indicates deficits in their *ethical acting*, as no account is seemingly taken of the complexity that older patients’ multiple diseases can present. The illness trajectory for a patient with heart failure is different from the one of cancer patients, or patients affected by frailty and/or dementia [33]. Being multi-diseased, older patients can follow all these trajectories at the same time, which may make their symptoms more difficult to assess. Therefore, it cannot be denied that many of them could benefit from being provided care in the ambulance, a space that older patients have described as containing advanced resources and competent staff who can provide the needed aid and safety in a vulnerable situation [34]. Thus, ACs’ tendency to leave older patients at home can be seen as an expression of ageism, signifying the discrimination against, and prejudicially stereotyping of, older people [35]. This can include a behavioural component, where ACs assume that all older people are generally vulnerable and weak, treating them as such, and thereby resulting in discrimination against them. The ACs’ actions may seem empathetic, but older patients have been shown to cherish their freedom [36]. Thus, regardless of bodily weakness, they often want to be involved in decisions concerning their lives. Then, ageist actions, despite being performed in a gentle manner, may make older patients feel ignored and objectified. Adding to this, older people may possess an inner strength, acquired throughout the various struggles of a long life, that helps them uphold their decision-making capability [37]. Consequently, many of them may be less vulnerable than their outer appearance indicates. ACs in this study seem to disregard this inner strength of older patients, which reveals some shortcomings in their *ethical reflection*, which may then have an impact on their *ethical actions.* In their defence, it can be said that they are aware of their lack of knowledge about multi-diseased older patients.

In situations of emergency care, i.e., when an older patient has suffered a cardiac arrest, ACs reveal a strong desire to protect themselves, by treating and transporting dying patients, not primarily for the benefit of the patients, but in order to follow their medical guidelines and prevent critique and disciplinary actions. Thus, when reflecting about their own role, they merely reflect on their own actions and not their values, which reveals a lack of *ethical reflection*. What seems to be needed here is the virtue of courage, that is, the middle course between cowardice and recklessness [38]. Nevertheless, ACs in this study expect their ability to make holistic assessments to grow with time and experience and develop their courage and ability to stand up for the patients’ will. This is congruent with the findings of an earlier study, highlighting that ACs’ trust in themselves can grow over the years and develop an inner security and confidence in their professional role [29]. In this study, ACs reflect on common collegial communication as being something valuable, one which prepares their actions and confirms their reasoning after a completed assignment. This aligns with an earlier study describing ACs’ work in dyadic teams to increase their confidence, broaden their experience and generate clarity on their way to the patient [39]. Consequently, well-functioning dyadic teams appear to be a prerequisite for developing *ethical competence* when caring for older patients with reduced decision-making ability.

Thus, in spite of elucidating the importance of the patient relationship, having developed an ethical competence to achieve a holistic and relational care in some respects, ACs in this study also appear to lack sufficient knowledge regarding the complexity of relational ethics, in terms of paternalism, shared decision-making and ageism, and of multi-diseased older patients. If they had a deeper understanding of these aspects, and consequently a more extensive ethical competence, one may anticipate that they would find it easier to cope with difficult situations when caring for older patients with reduced decision-making capacity.

### Limitations

This study has some limitations. First, it can be considered a limitation in trying to capture the phenomenon of ethical competence when caring for older patients with reduced decision-making ability in a limited healthcare context such as ambulance care. The transferability of the results to other contexts should therefore be done with some caution, also considering the relatively limited sample size from one region of one country. However, there is reason to assume that the results are transferable to contexts with similar demographics, resources, healthcare, and ambulance services with registered nurses with similar level of education and competence as Swedish nurses. Second, ethical competence is multidimensional and there is disagreement about which dimensions should be included in the competence. However, the choice of a deductive approach and use of a definition of ethical competence in the context of nursing practice should have increased the possibility to compare ethical competence between healthcare contexts in the future if studies replicate the same approach. The use of a well-described vignette in the interviews also increases the replicability of the study. Third, the first author is a registered nurse with experience in nursing home contexts, thus lacking experience within ambulance care. This lack of pre-understanding may have influenced the analysis, as preunderstanding is an asset that helps researchers to understand the data [40]. However, the two other authors are experienced ACs, and, in critical discussions performed within the research group as a whole, the common pre-understanding was broadened and enrichened by the first author’s experiences from another care context that concerns older patients. The conscious reflective stance in the data analysis is deemed to have strengthened the validity of the study. Further, engaging in critical discussions with other researchers has also contributed to the trustworthiness of this study [23]. Finally, the reliability of the study is judged to have been strengthened by the accuracy and reporting of the research process and the vignette, and the use of a definition of ethical competence in the data analysis.

## Conclusions

ACs possess an *ethical competence* in terms of *ethical knowledge* that highlights the need of an interpersonal relationship with the older patients. To establish this relationship, they use *ethical sensitivity* to interpret the patients’ needs. Doing this, they are aware of the importance of *ethical behaviour*, that is, to act respectfully and provide the time needed for listening and interaction. However, they fail to see their gut feeling, upon which they rely in precarious situations, as a professional *ethical competence*, which might hinder them from reacting to unethical ways of working. Further, they lack *ethical reflection*, for instance, regarding the benefits and disadvantages of paternalism, which reduces their ability to perform *ethical decision-making*. Another aspect of reduced *ethical knowledge* is their ageist approach to older patients, shown by their opinion that older patients feel best when being cared for in their home, which has consequences for their *ethical acting*. Finally, ACs show deficiencies in regards to their *ethical reflections*, as they reflect merely on their own actions, rather than on their values.

### Electronic supplementary material

Below is the link to the electronic supplementary material.

Supplementary Material 1

## Data Availability

Datasets used in the current study are available in Swedish from the corresponding author upon reasonable request.

## List of abbreviations

AC: Ambulance clinician
DNAR: Do Not Attempt Resuscitation order
